# Fast and Sensitive Bioanalytical Method for the Determination of Deucravacitinib in Human Plasma Using HPLC-MS/MS: Application and Greenness Evaluation

**DOI:** 10.3390/molecules28145471

**Published:** 2023-07-17

**Authors:** Pottabattula Mahesh, M. Akiful Haque, Baher I. Salman, Tarek S. Belal, Adel Ehab Ibrahim, Sami El Deeb

**Affiliations:** 1 Department of Pharmaceutical Analysis, Anurag University, Venkatapur, Ghatkesar Rd., Hyderabad 500088, Telangana, India; mahi_pharmadbm@yahoo.co.in (P.M.); akifulhaquepharmacy@anurag.edu.in (M.A.H.); 2 Pharmaceutical Analytical Chemistry Department, Faculty of Pharmacy, Al-Azhar University, Assiut Branch, Assiut 71524, Egypt; bahersalman@azhar.edu.eg; 3 Pharmaceutical Analytical Chemistry Department, Faculty of Pharmacy, University of Alexandria, Alexandria 21521, Egypt; tbelaleg@yahoo.com; 4 Pharmaceutical Analytical Chemistry Department, Faculty of Pharmacy, Port-Said University, Port-Said 42511, Egypt; adel@unizwa.edu.om; 5 Natural and Medical Sciences Research Center, University of Nizwa, Birkat Al Mauz, Nizwa 616, Oman; 6 Institute of Medicinal and Pharmaceutical Chemistry, Technische Universitaet Braunschweig, Beethovenstr. 55, 38106 Braunschweig, Germany; 7 Institute of Pharmacy, Freie Universität Berlin, Queen-Luise-Strasse 2 and 4, 14195 Berlin, Germany

**Keywords:** deucravacitinib, HPLC-MS/MS, psoriasis, human plasma

## Abstract

Plaque psoriasis is a common, long-lasting illness that affects the immune system and causes significant negative impacts on a patient’s physical health, well-being, and ability to work effectively. Deucravacitinib (DEU) is the first oral medication used in the treatment of plaque psoriasis, a chronic skin condition that causes red, scaly patches on the skin. DEU is a type of medication called an oral Janus kinase (JAK) inhibitor, which works by blocking specific enzymes that play a role in the inflammation and immune response associated with psoriasis. Therefore, a quick, easy, novel, reliable, sensitive, and straightforward liquid chromatography-tandem mass spectrometry (LC-MS/MS) approach was used to analyze DEU in plasma samples. The LC-MS/MS method for the determination of DEU in human plasma was based on using trimethoprim as an internal standard (IS). The separation of DEU and IS was carried out via liquid–liquid extraction (LLE). The extract was then subjected to the chromatographic system separation using the ACE-C18 column (4.6 × 100 mm, 5 µm). The mobile phase employed consisted of methanol and a solution of 2 mM ammonium formate (80:20 *v*/*v*, respectively). The flow rate used was set at 0.9 mL min^−1^. The creative strategy was performed by running an ABSCIEX API 4000 mass spectrometer with an electron spray ionization source in multiple reaction monitoring (MRM) mode. The ion transitions *m*/*z* 426.3 → 358.2 were used for DEU quantitation, while the ion transitions *m*/*z* 291.1 → 261.1 were used for trimethoprim quantitation. The accuracy, precision, linearity, recovery, and selectivity of DEU were deemed acceptable when validated for a concentration range between 0.500 and 601.050 ng/mL, utilizing a weighting factor of 1/x^2^.

## 1. Introduction

Psoriasis is a commonly known pathological skin disease that has a characteristic autoimmune condition [1]. The inflammatory skin state is chronic and is distinguished by epidermal hyperplasia and a thick and scaling character, where the immune cells are extensively infiltrated [1]. Psoriasis prevalence is high and affects about 2–3% of the European and United States population [2,3]. With more than 125 million patients suffering from psoriasis worldwide, the consequences are expected to represent a substantial economic and health problem [2]. Psoriasis is clinically indexed, according to the presence of pustules, into two groups—non-pustular psoriasis and pustular psoriasis [4]. Plaque psoriasis, also known as psoriasis vulgaris, is the most frequent form of this disease belonging to the non-pustular psoriasis type, which accounts for about 80–90% of diagnosed patients [2,4]. Psoriasis plaques show up as raised, inflamed, and textured patches of skin that will be bothersome and difficult. On Caucasian skin, plaques ordinarily show up as raised, ruddy patches secured with a shimmering white buildup of dead skin cells or scale [5]. This infection has a strong genetic basis that is complex and difficult to understand, with a level of agreement of approximately 60% in identical twins [6]. Psoriasis vulgaris is not a genetically homogeneous disease and it appears that distinct clinical subtypes of the disease are influenced by different genetic components [5,6]. The development of several comorbidities associated with psoriasis is high. For instance, psoriatic arthritis is common for 30% of psoriasis patients [7]. Cardiovascular diseases and mental disorders are other risky comorbidities [2,7]. The morbidity rates of these diseases are substantial.

For decades now, the clinical treatment of psoriasis has been established on topical treatments [1] and phototherapies [2]. Other systemic treatments were previously established on biological and non-biological molecules. The non-biological molecules, like methotrexate, ciclosporin, and others, have several unavoidable side effects, such as kidney failure, fetal abnormalities, and others [8]. With more than 11 approved molecules, biological regimens have a better tolerability than the previously described non-biological ones. They can reduce the inflammation through targeting the immune cells responsible for the disease; however, their selectivity is a major concern. Biological treatments can affect the whole immune response, hence severe infections are serious side effects [9]. Moreover, biological molecules have economic drawbacks. When compared to the small drug molecules, they are more difficult to synthesize, more costly, and more difficult to administer (parenteral routes instead of oral) [10]. Therefore, the development of novel, more selective, safer, low cost, and effective oral regimens has been a challenge.

**Figure 1:**
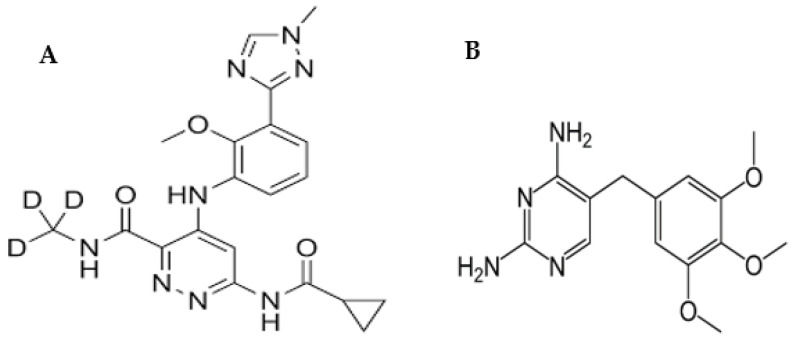
Chemical structures of DEU (**A**) and TMP (**B**).

In September 2022, the United States FDA approved the first of its class oral drug, (DEU) [11]. DEU (Figure 1) was approved as a first-line oral and selective tyrosine kinase inhibitor [12,13]. DEU is a novel lingual small molecule that selectively impedes TYK2 by binding exclusively to the TYK2-regulated pseudo-kinase (JH2) domain (allosteric inhibition). DEU, like other JAK inhibitors, does not bind to the kept active domain (competitive inhibitor) and is therefore significantly selective for TYK2 over other JAKs [14,15]. Therefore, DEU can be used to treat adults with moderate-to-severe plaque psoriasis who did not respond to systemic medication or phototherapy [12,15].

Being the first oral pill indicated for psoriasis once daily, novel methodologies should be required to monitor the drug in plasma to evaluate its pharmacokinetics and pharmacodynamics. The determination of drugs after oral tablet and capsule dosage forms in human plasma is important for several reasons. First of all, the determination of oral solid dosage forms in human plasma is important for therapeutic drug monitoring. By measuring the concentration of a drug in a patient’s plasma after oral administration, the dosage could be adjusted to ensure that the drug is effective and safe. Some recent reports have studied the pharmacodynamics and pharmacokinetics of DEU after oral administration for different reasons [16,17]. Meanwhile, another reason for establishing human plasma concentration studies is because it is an important tool for assessing the bioequivalence as represented in the rate and extent of drug absorption from different formulations. The ICH guidelines for bioequivalence studies of newly developed oral solid dosage forms emphasizes the crucial importance of comparing the drug concentration in plasma for generic and innovator pharmaceutical products as part of its new drug registration [18]. Moreover, to understand how drugs are absorbed, distributed, metabolized, and eliminated in the body, the drug concentration must be followed in human plasma in a pharmacokinetic study [19].

To our knowledge, not a single analytical method has yet been reported for the determination of DEU in either plasma, biological fluids, or marketed dosage forms. The proposed study should be the first to report and validate an analytical methodology for the estimation of the drug under study using the LC-MS/MS technique. The proposed LC-MS/MS strategy is a selective, ultra-sensitive, and accurate approach with high repeatability and reliability. The main research activity was aimed at developing a bioanalytical design that is suitable for the quantification of DEU in human plasma, for the first time, covering a wide range of linearity.

One of the main advantages of LC when coupled with MS/MS detection is its high selectivity and sensitivity [20]. However, the major drawback of MS/MS detection is the low reproducibility of results owing to matrix effects. Therefore, the use of an internal standard (IS) is common in LC-MS/MS to correct for some factors such as ionization efficiency, injection volume, and matrix effect variations. The use of IS is a perfect strategy for improving the precision of the analysis as well. The choice of an effective IS should be based on the physicochemical properties of the analyte of interest to correct for any changes in ionization efficiency that may result from matrix effects. As seen in Figure 1, DEU is a free base with basic functional groups available for the extraction and ionization of DEU. The non-polar nature makes it prone to problems with matrix effects. Therefore, trimethoprim (TMP) (Figure 1) was chosen as an internal standard (IS) to correct for matrix effects and ensure that the IS-normalized matrix coefficients are within acceptable limits. Both drugs, DEU and TMP, have low water solubilities (lipophilic nature). TMP was also chosen because both analytes have basic characters as shown from their basic functional groups (pKa values of 7.1 and 11.0 for TMP and DEU, respectively) [21,22]. The developed method was then assessed for its ecological greenness using different metrics in order to enrich its analytical value [23].

## 2. Results

### 2.1. Mass Spectrophotometry

**Figure 2:**
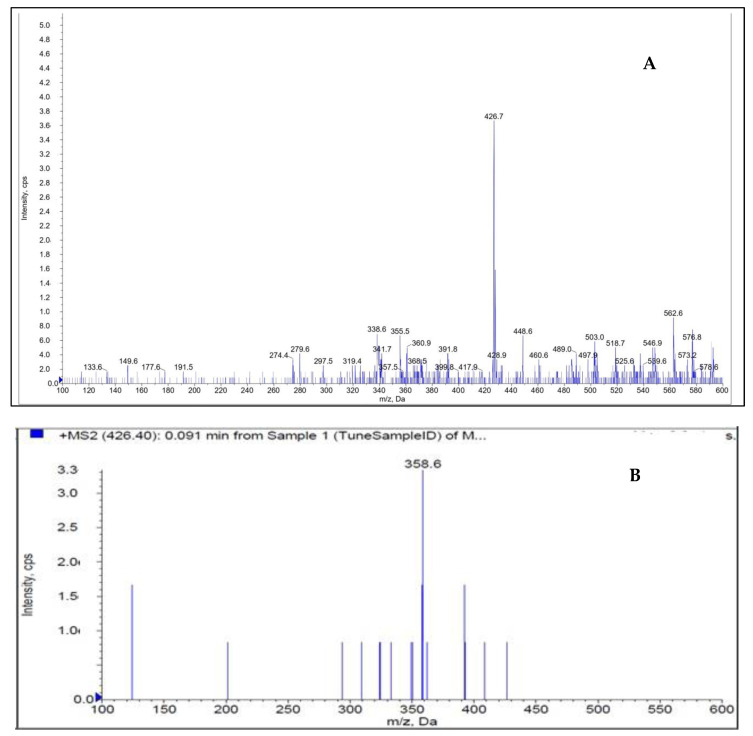
Parent ion (**A**) and daughter ion (**B**) spectra of [M + H]+ of DEU.

Figure 2 shows how the mass characteristics for the compounds with good performance were optimized in positive ionization mode. Data from the MRM mode were evaluated to improve selectivity [24,25,26]. The *m*/*z* values of the protonated form analyte and IS, [M + H]^+^ were 426.7 and 291.1 (Q1 mass), respectively. The daughter masses were determined to be 358.6 and 261.1 (Q3 mass), respectively (Figure 2).

### 2.2. The Development of the Creative Approach

To obtain the requisite separations, a series of studies was carried out utilizing formate and acetate buffers of varying pH. Based on the findings, ammonium formate was chosen as the buffer and methanol was used as the organic solvent. Various buffer and methanol ratios were explored and, finally, the methanol: buffer (80:20, *v*/*v*) was chosen as the optimized mobile phase because it eluted an elevated peak with favorable features for DEU and TMP as IS. The devised approach (Table 1 and Table 2) produced a symmetric peak with a retention period of 1.56 min for DEU and 1.34 min for TMP and met all peak attributes as specified by USP guidelines [27].

### 2.3. Chromatography

The measuring of the DEU in human plasma was observed using the LLOQ of DEU compared with free plasma samples (Supplementary Material Figure S1) and LLOQ of TMP (IS) (Supplementary Material Figure S2). The final optimized chromatographic parameters for the estimation of DEU in plasma samples were observed in Table 2.

### 2.4. Method Validation

#### 2.4.1. Specificity

Selectivity of the creative strategy was performed by checking blank plasma (without spiking with DEU) from six individual blood donor lots. Each blank plasma was processed against each LLOQ and analyzed. Interference in blanks at the analyte retention time (RT) was less than 20% of the area of the respective LLOQ. Interference in the blank at the IS retention time was less than 5% of the respective internal standard IS area (Supplementary Material Table S1).

#### 2.4.2. Linearity

To define the range of DEU concentrations that can be tested with the creative approach, we collected and analyzed eight different sets containing DEU concentrations ranging from 0.5 to 601.05 ng mL^−1^, utilizing a weighting factor of 1/x^2^ (Table 3). The use of this approach in MS detection techniques is very effective for improving linearity, accuracy, and precision, especially in low abundance analytes [28]. The area ratios obtained for each concentration were plotted against the concentration of DEU. The points were linearly fitted by least-squares regression analysis and constant proportions with minimal data variance were observed. The 75% calibration standards must be within 85–115% of nominal concentration except for calibration standard (STD1), where it can be within 80–120% of the nominal concentration (i.e., LLOQ). The mean correlation coefficient (r^2^ value) obtained was greater than 0.9941 (Table 3). Hence, the DEU can be easily estimated with the present system within this concentration. The lower limit of quantification (LLOQ) for DEU is 0.500 ng mL^−1^.

#### 2.4.3. Accuracy and Precision

Assay accuracy was defined as the ratio of the mean assay value to the actual value collected and was expressed as a percentage. As shown in Table 4, the accuracy results range from 95.98% to 108.59%. The impact captured is shown in Table 4. Based on the results obtained, the accuracy ranged from 3.03 to 5.34%.

Precision was established for the three quality control standards for six sets of QC specimens within the same day (intra-day) and between three different days (inter-day). Intra-day and inter-day precisions were found to be consistent against a single linear curve using three concentrations of LQC, MQC, and HQC (1.444, 240.733, 456.798 ng mL^−1^).

The run sensitivity of DEU studied at LLQC 0.500 ng mL^−1^ was 96.07% recovery with a reasonable RSD (Table 4).

#### 2.4.4. Recovery

The extraction recoveries for DEU at low (1.444 ng/mL), medium (240.733 ng/mL), and high (456.798 ng/mL) plasma concentrations with six replicate injections each showed 60.89%, 66.82%, and 68.36%. The overall recovery of DEU was found to be 65.35%. The recovery of DEU was found to be appropriate, precise, and reproducible (Table 5).

#### 2.4.5. Matrix Effects

Six individual lots of human plasma were utilized, extracted blanks and post-extracted blanks were prepared from each plasma, and LQC and HQC levels were analyzed. No significant matrix ions were observed. At LQC and HQC concentrations, the internal standard matrix factor was found to be from 0.96 to 1.04 (Table 6).

#### 2.4.6. Stability

The stability of the analytes after extraction from human plasma was assessed using two different quality control standards (LQC and HQC). The stability of the standards under room temperature (for 12 h), under auto-sampler nominal conditions kept cold at 10 °C (for 24 h), and under refrigerator storage at 2–8 °C (for 25 h), was determined. The recovery percentages of the two standards’ concentrations were calculated and are included in Table 7.

### 2.5. Evaluation of Method’s Analytical Greenness

One of the main current focuses of analytical chemists is ecological safety and sustainability. The routine use of the analytical methodologies during the research and quality control activities could generate substantial amounts of persistent environmental hazards. To illustrate, on a daily basis, a single piece of equipment of a conventional HPLC could generate up to 0.5 L of organic waste [29]. For this reason, the assessment of the ecological impact of newly established analytical methodologies became a requisite step during their development and evaluation. During the past decade, several metrics have been reported in detail for such greenness assessments. The analytical eco-scale [30] was one of the earliest and most highly cited metrics. Thereafter, more efficient metrics were developed including the green analytical procedure index (GAPI) [31], the AGREE metric [32], RGB 12 algorithms [33,34], and hexagon [35].

**Figure 3:**
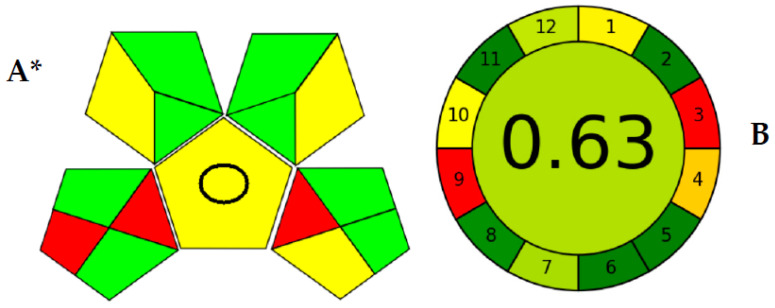
Greenness evaluation of the proposed method using GAPI (**A**) and AGREE (**B**) metrics. * GAPI: The pentagrams representing sampling procedure (**lower left**), sample preparation (**upper left**), reagents used (**upper right**), and instrumentation (**lower right**) and the central pentagram including a circle indicating a quantification method for analysis.

Among those metrics, GAPI [31] has gained plenty of attention as being one of the earliest assessment methods that considered the whole analytical procedure. The GAPI pictogram is composed of five pentagons. This means that GAPI considered five main processes alongside the analytical methodology, including the sampling procedure, sample preparation, reagents used, and instrumentation. Then, those pentagons comprise 15 different areas, each representing a step that falls within the analytical procedure. The exact analytical procedure was considered, besides the generated waste and its treatment strategy. Figure 3 shows the assessment of the validated method using GAPI and AGREE. As shown (Figure 3A), the GAPI pictogram has only three red zones, which represent the off-line sampling and transportation of the sample in the lower left pentagon. Another red zone is found in the lower right pentagon, representing the higher energy utilized by LC-MS/MS instrumentation.

Meanwhile, the AGREE pictogram has a clockwise shape, the perimeter of which is divided into 12 areas. AGREE was built mainly on the twelve principles of green analytical chemistry (GAC) [32]. Therefore, each green analytical chemistry is represented by a zone within the AGREE pictogram’s perimeter. The core part of the pictogram has a figure from 0.00–1.00. As the ecological sustainability of the developed methodology is higher, the closer this number is to 1.00. As shown in Figure 3B, the AGREE assessment showed only two red zones within the pictogram perimeter for the off-line analysis and the energy utilized by the analytical equipment. However, AGREE has the advantage of considering the number of analyses performed per hour (the method’s throughput), which was not considered by GAPI earlier. The large analytical throughput, the nano-sized sample, the minimal amounts of consumed reagents, and waste generated proved the enhanced ecological impact.

Another advantage of the proposed methodology is that it uses methanol as an organic modifier, which is much more benign than acetonitrile [36], although the fraction of the organic modifier, methanol, in the mobile phase was 80%. However, the use of a selective MS/MS detector enabled the selective identification of DEU and TMP in a short chromatographic run that did not exceed 1.6 min. That was reflected in the amount of organic modifier used not exceeding 1.1 mL, only in a fast procedure and with a higher analytical yield.

## 3. Materials and Methods

### 3.1. Instrumentation

A mass spectrometer SCIEX API 4000 (from AB Sciex LLC, Framingham City, MA, USA) with Shimadzu prominence LC (software Version-Analyst 1.6.3) interfaced via Turbo ion spray was used for the study. The chromatographic method was optimized using the ACE C18 column (100 × 4.6 mm, 5 μm).

### 3.2. Materials

DEU and TMP (Internal Standard) were obtained as a gift sample from Dr. Reddy’s Laboratories (Telangana, India). Methanol (HPLC grade), ammonium formate (AR grade), methyl tertiary butyl ether (MTBE) (AR grade), and HPLC water were purchased from Merck (Darmstadt, Germany). Human (K2 EDTA as an anticoagulant) plasma was obtained from the Om blood bank (Pune, Maharashtra, India). The study protocol was reviewed and approved by the Aavishkar Ethics Committee (Tiswadi City, Goa, India).

### 3.3. Chromatographic Conditions

The mobile phase was composed of methanol and 2 mM ammonium formate (80:20, *v*/*v*, respectively) which was used for the separation of analyte from the internal standard as well as extracted samples at a flow rate of 0.9 mL min^−1^. Quantitation of the separated components was performed in a mass spectrometer in positive ion mode. A highly sensitive and selective, rapid, MRM (Multiple Reaction Monitoring) strategy was created and validated for the quantification of DEU in human plasma utilizing an isocratic elution with 5 µL injection on tandem mass spectrometry. This innovative approach is based on liquid–liquid phase extraction with selective and rapid TMP as an IS. Sample 100 ng mL^−1^ (in 90% methanol in water) was prepared and used for the tuning of mass parameters.

### 3.4. Preparation of Standard Solutions

Of each DEU and TMP standard, 5.0 mg was weighed and dissolved in 5.0 mL methanol to obtain 1 mg mL^−1^ primary stock solutions, which were stored in a refrigerator (2–8 °C). The linear graph calibrators and the quality control standards were prepared using those primary stock solutions. A working solution of IS (500 ng mL^−1^) was obtained by dilution using a mixture of methanol: water (80:20, *v*/*v*).

### 3.5. Linear Graph and Control Samples

The samples were spiked with 0.980 mL of control plasma with the standard dilution of 0.02 mL dilution of the analyte. A set of eight non-zero standards ranging from 0.500 to 601.050 ng mL^−1^ of DEU were prepared for the linear graph. To determine the precision and accuracy, sample solutions (0.2 mL each) were prepared by spiking the control human plasma (9.8 mL each). The final concentrations for QC samples for DEU were 0.5 ng mL^−1^ (LLOQ), 1.444 ng mL^−1^ (LQC), 240.733 ng mL^−1^ (MQC), and 456.798 (HQC) ng mL^−1^.

### 3.6. Sample Processing

Two main techniques were considered during the method development stage—liquid–liquid extraction (LLE) and solid phase extraction (SPE). LLE was chosen for its simplicity and cost effectiveness compared to SPE. Moreover, the recovery% of analytes using LLE is high and can be optimized. The choice of solvent used for LLE was based mainly on the hydrophobicity of the target analytes. In the proposed study, the drugs targeted were more lipophilic. Therefore, a nonpolar solvent was selected to extract the nonpolar compounds from aqueous plasma samples. Hexane, cyclohexane, and methyl tertiary butyl ether (MTBE) were tried. However, the best efficiency of extraction was obtained using MTBE. In the proposed study, the LLE method [24,25] was used to isolate DEU and TMP as IS from human plasma. A 0.100 mL sample (K2 EDTA plasma) was aliquoted into Ria (polypropylene) vials. Another 0.050 mL of IS working solution was added to all samples except the blank and vortexed ones. Then, 0.100 mL of 2 mM ammonium formate was added to all samples and vortexed and 1.000 mL of methyl tertiary butyl ether (MTBE) was added to all samples. All samples were vortexed at 2500 rpm for 10 min. The supernatant was separated into a fresh Ria vial. Samples were evaporated at 40 °C under nitrogen gas until dryness. The samples were then reconstituted with a 0.500 mL mobile phase and vortexed for 3 min. Samples were transferred to an autosampler vial for analysis and 5 μL of the sample was injected into a chromatographic system with MS-MS detection.

### 3.7. The Validation Process of Innovated Strategy

The method has been validated under FDA guidance [37].

#### 3.7.1. Selectivity

At least six individual donor lots of human plasma were used for analyses of blank samples for selectivity. One blank and one lower limit of quantitation (LLOQ) were processed from each plasma lot and analyzed for interference and selectivity.

#### 3.7.2. Precision and Accuracy of the Creative Strategy

According to FDA guidance [37,38], a minimum of three concentrations in the range of expected concentrations under a calibration curve should be used for accurate results. To estimate the assay accuracy, six replicates containing analyte at a minimum of three different quality control (QC) levels were analyzed. This analysis ensures that the mean value falls within 15% of the actual concentration, providing reliable data for further decision-making. By adhering to these guidelines, both precision and accuracy can be ensured in the calibration process while maintaining compliance with regulations.

To achieve the required precision, it is essential to perform a minimum of five determinations per concentration at no less than three varying concentration levels. The coefficient of variation (CV) for each concentration level must not surpass 15%, except for lower limit of quantification (LLOQ) measurements, where a maximum of 20% CV is acceptable. Following these standardized procedures will foster the production of reliable data and reinforce the robustness of the calibration process. Moreover, it will guarantee our adherence to regulatory requirements and promote confidence in the analytical results generated.

Recovery of the analyte does not need to be 100% but the degree of recuperation of an analyte and the inner standard ought to be reliable, exact, and reproducible. Recovery tests ought to be performed by comparing the expository results for extricated tests at three concentrations (low, medium, and maximum concentrations) with un-extracted/aliquot guidelines that speak to 100% recuperation.

#### 3.7.3. Standardization and Calibration Graph

The curve for calibration was measured using samples that were produced in the same biological medium by spiking the matrix with specified analyte concentrations. The range of expected analytical results and the type of the analyte/response connection determine how many standards are required when building a calibration curve. Standard levels ought to be determined by the concentration range anticipated in a specific investigation. A calibration curve included a blank sample, a zero sample, and six-to-eight non-zero specimens that spanned the predicted range, including LLOQ. A blank sample is a matrix specimen treated without an internal standard, whereas a zero sample is a matrix sample generated with an internal standard.

#### 3.7.4. Stability Study of DEU

To evaluate the stability of the DEU in samples of plasma under different circumstances that could exist during sampling, stability studies were carried out. Six different samples from each level were used to perform the LQC and HQC levels of the stock solution stability, autosampler (processed sample) stability, and extracting stability tests. By contrasting the recoveries of the quality control samples under the various stability settings with those of freshly created samples, the stability of the QC samples was examined. If the average concentration at each QC level was less than 15% and the RSD% did not go over 15%, the samples were considered stable.

## 4. Conclusions

Considering the information provided within the proposed study, it can be said that a novel method is approved for the liquid–liquid extraction-based detection of deucravacitinib in human plasma at concentrations between 0.500 and 601.050 ng mL^−1^. In this concentration range, the precision and accuracy are within the appropriate limits. Estimated recoveries are deemed appropriate for the current LQC, MQC, and HQC processing techniques. It turned out that recoveries are due to matrix effects of ~60% but they are consistent and reproducible. The drug was also discovered to be stable when subjected to the effects of wet extraction, auto-sampler, and stock solution stability. The proposed method showed excellent accuracy, precision, recovery, and sensitivity within the shortened run period. To date, no analytical approaches had been reported for the quantification of DEU in biological fluids. The selective LC-MS/MS strategy was considering the first analytical method to develop a selective, ultra-sensitive, and accurate approach with high repeatability and reliability.

## Figures and Tables

**Table 1:** Optimization data for DEU and TMP using the devised approach.

Name	Q1 Mass (amu)	Q3 Mass (amu)	Dwell (ms)	DP (V)	EP (V)	CE (V)	CXP (V)
Deucravacitinib	426.7	358.6	200	110	10	32	10.30
Trimethoprim	291.1	261.1	200	110	10	32	10.30
CUR (psi)	CAD (psi)	Ion Spray Voltage (V)	TEM (°C)	GAS 1 (psi)	GAS 2 (psi)	Scan Type	Polarity
20	10	5500	500	40	40	MRM	Positive

**Table 2:** Optimized method development parameters for DEU determination.

Parameter	Details
Column	ACE C_18_ (100 × 4.6 mm, 5 µm)
Pump mode	Isocratic
Column temperature	Ambient
Flow rate	0.9 mL min^−1^
Injection volume	5.0 µL
Run time	3.0 min
Detector	Tandem mass spectrometry (MRM mode)
Mobile phase	Methanol: 2 mM ammonium formate (80:20, *v*/*v*)

**Table 3:** Back-calculated concentrations of calibrant samples for DEU in human plasma (*n* = 3).

Calibration Standard ID	Nominal Conc. (ng mL^−1^)	Calculated Conc. (ng mL^−1^)
STD1	0.500	0.488
STD2	1.001	1.082
STD3	2.502	2.271
STD4	10.007	10.447
STD5	50.037	43.694
STD6	200.150	264.329
STD7	540.945	468.146
STD8	601.050	687.353
Slope		0.0362
Intercept		0.000404
Correlation Coefficient (r^2^)		0.9941

**Table 4:** Accuracy, precision, and sensitivity results for DEU determination.

Standard/Concentration	LQC (1.444 ng mL^−1^)		MQC (240.733 ng mL^−1^)		HQC (456.798 ng mL^−1^)		Sensitivity LLQC (0.4803 ng mL^−1^)	
	R%	RSD	R%	RSD	R% *	RSD **	R%	RSD
Accuracy	108.14	5.34	108.59	3.03	95.98	3.65	96.07	9.5
Intra-day Precision	108.43	5.79	108.58	3.29	95.78	3.50		
Inter-day Precision	105.08	6.25	106.94	3.22	97.41	3.15		
*n*	*n* = 6							

* Recovery% = calculated concentration/Actual concentration × 100. ** RSD: Relative standard deviation.

**Table 5:** Recovery results for DEU determination.

QC Sample	Recovery ± RSD% (*n* = 6)
LQC	60.89 ± 1.50
MQC	66.82 ± 2.02
HQC	68.36 ± 1.88
Average ± RSD%	65.35 ± 1.50

**Table 6:** Matrix factor of internal standard (*n* = 6).

Parameter	DEU	IS	IS Normalization
Matrix factor	0.94	0.95	0.99
RSD	2.37	4.20	2.89

**Table 7:** The stability results of DEU standards under different conditions (*n* = 6).

Standard (ng/mL)	Room Temperature Stability		Auto-Sampler Stability		Refrigerator Stability	
	R%	RSD	R%	RSD	R% *	RSD **
LQC (1.444)	107.96	1.47	108.07	2.36	107.63	2.50
HQC (456.798)	102.66	0.60	102.58	0.51	98.96	2.12

* Recovery% = calculated concentration/Actual concentration × 100. ** RSD: Relative standard deviation.

## Data Availability

Not applicable.

## Supplementary Materials

The following supporting information can be downloaded at: https://www.mdpi.com/article/10.3390/molecules28145471/s1, Figure S1: (a) Deucravacitinib, extracted blank plasma chromatogram, and (b) Deucravacitinib, extracted LLOQ chromatogram Not applicable; Figure S2: (a) Trimethoprim (IS), extracted blank plasma chromatogram, (b) Trimethoprim (IS), extracted LLOQ chromatogram; Table S1: Calculation of% interference in blank.
