# Supplementary material for: Fast and Sensitive Bioanalytical Method for the Determination of Deucravacitinib in Human Plasma Using HPLC-MS/MS: Application and Greenness Evaluation
Source: Molecules. 2023 Jul 17;28(14):5471. doi: 10.3390/molecules28145471 (PMC10384560; doi:10.3390/molecules28145471)
Supplement: Supplementary file 1 [file molecules-28-05471-s001.zip › molecules-2484619-supplementary.pdf]

Supplementary Materials

Fast and Sensitive Bioanalytical Method for the Determination of Deucravacitinib in Human Plasma using HPLC-MS/MS; Application and Greenness Evaluation

Pottabattula Mahesh [1], M. Akiful Haque [1], Baher I. Salman [2], Tarek S. Belal [3], Adel Ehab Ibrahim [4,5], Sami El Deeb [6,7,*]

1 Department of Pharmaceutical Analysis, Anurag University, Venkatapur, Ghatkesar Rd., Hyderabad 500088, Telangana, India; mahi_pharmadbm@yahoo.co.in (P.M.); akifulhaquepharmacy@anurag.edu.in (M.A.H.)
2 Pharmaceutical Analytical Chemistry Department, Faculty of Pharmacy, Al-Azhar University, Assiut Branch, Assiut 71524, Egypt; bahersalman@azhar.edu.eg
3 Pharmaceutical Analytical Chemistry Department, Faculty of Pharmacy, University of Alexandria, Alexandria 21521, Egypt; tbelaleg@yahoo.com
4 Pharmaceutical Analytical Chemistry Department, Faculty of Pharmacy, Port-Said University, Port-Said 42511, Egypt; adel@unizwa.edu.om
5 Natural and Medical Sciences Research Center, University of Nizwa, Birkat Al Mauz, Nizwa 616, Oman
6 Institute of Medicinal and Pharmaceutical Chemistry, Technische Universitaet Braunschweig, Beethovenstr. 55, 38106 Braunschweig, Germany
7 Institute of Pharmacy, Freie Universität Berlin, Queen-Luise-Strasse 2 and 4, 14195 Berlin, Germany
* Correspondence: s.eldeeb@tu-braunschweig.de

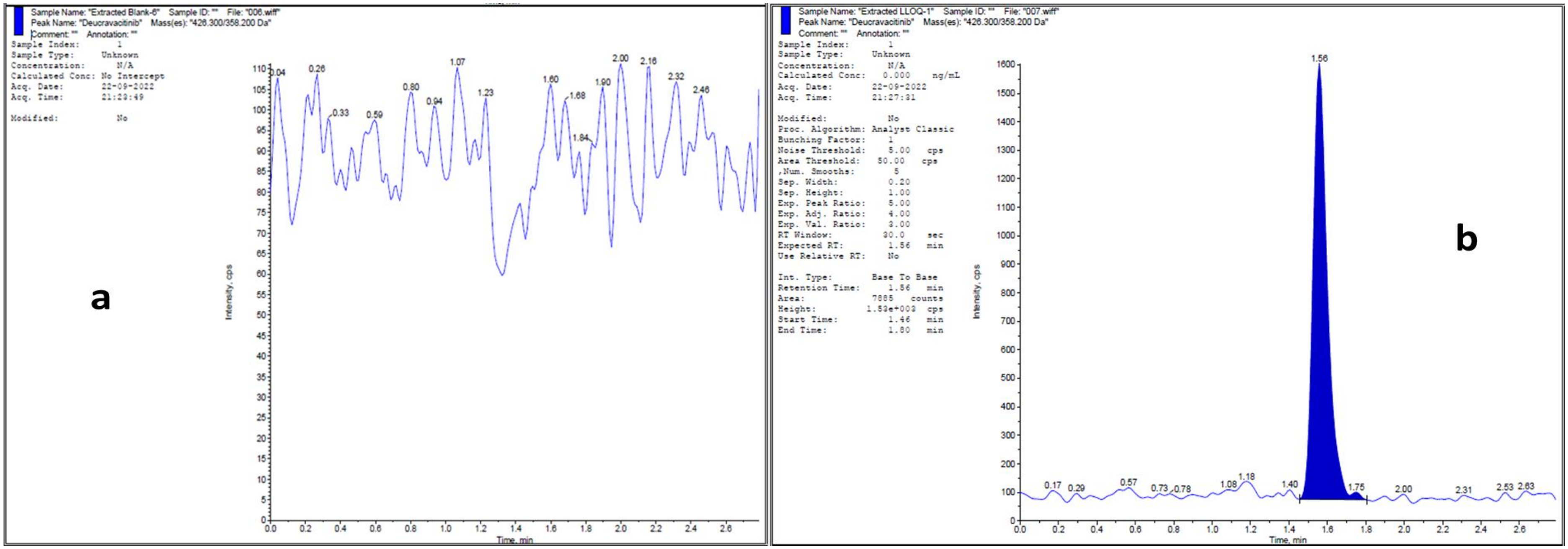

Figure S1: (**a**) Deucravacitinib, extracted blank plasma chromatogram, and (**b**) Deucravacitinib, extracted LLOQ chromatogram.

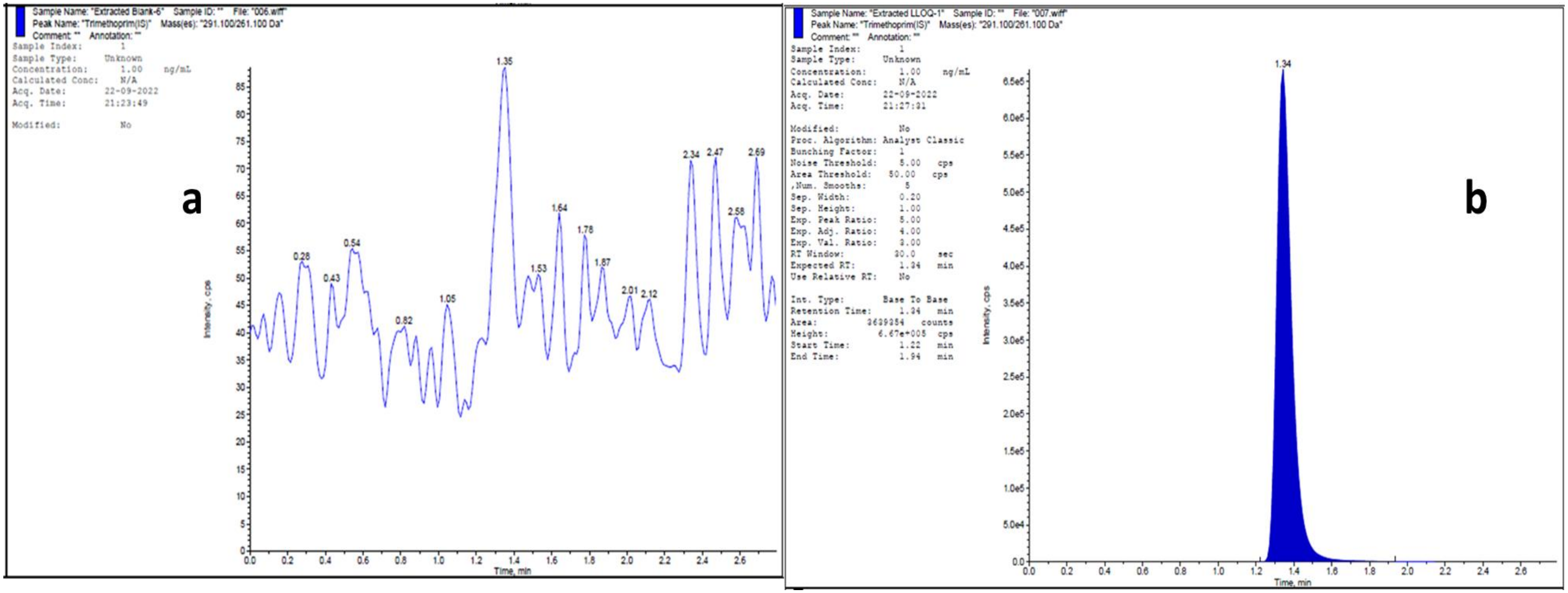

Figure S2: (**a**) Trimethoprim (IS), extracted blank plasma chromatogram, (**b**) Trimethoprim (IS), extracted LLOQ chromatogram**.**

Table S1: Calculation of % interference in blank

<table>
<tr><th>Sample Name</th><th>Area observed at RT of Analyte</th><th>Area observed at RT of IS</th><th>% Interference at analyte RT</th><th>% Interference at IS RT</th></tr>
<tr><td>Blank</td><td>183</td><td>385</td><td rowspan="2">2.32</td><td rowspan="2">0.01</td></tr>
<tr><td>LLOQ</td><td>7885</td><td>3639354</td></tr>
<tr><td>Blank</td><td>259</td><td>376</td><td rowspan="2">3.26</td><td rowspan="2">0.01</td></tr>
<tr><td>LLOQ</td><td>7938</td><td>3716553</td></tr>
<tr><td>Blank</td><td>286</td><td>0</td><td rowspan="2">3.61</td><td rowspan="2">0.00</td></tr>
<tr><td>LLOQ</td><td>7919</td><td>3755307</td></tr>
<tr><td>Blank</td><td>0</td><td>118</td><td rowspan="2">0.00</td><td rowspan="2">0.00</td></tr>
<tr><td>LLOQ</td><td>9965</td><td>3810218</td></tr>
<tr><td>Blank</td><td>249</td><td>293</td><td rowspan="2">3.16</td><td rowspan="2">0.01</td></tr>
<tr><td>LLOQ</td><td>7888</td><td>3813922</td></tr>
<tr><td>Blank</td><td>0</td><td>0</td><td rowspan="2">0.00</td><td rowspan="2">0.00</td></tr>
<tr><td>LLOQ</td><td>7814</td><td>3978417</td></tr>
</table>
